# SBCloud: A Cloud-Based Platform for Structure Determination and Data Storage

**DOI:** 10.1063/4.0001001

**Published:** 2025-10-27

**Authors:** Jason M Key, Benjamin Eisenbraun, Peter A Meyer, Piotr Sliz

**Affiliations:** 1 Harvard Medical School, Boston, MA, USA; 2 Boston Children's Hospital, Boston, MA, USA

## Abstract

Structural biology generates vast amounts of data, particularly in cryo-electron microscopy (cryo-EM), cryo-electron tomography (cryo-ET), and X- ray diffraction. Modern detectors produce many terabytes of data per day and require resource-intensive processing, often with specialized hardware. This data deluge creates significant challenges for storage and timely analysis. High-performance data storage systems are required for continuous data collection and processing, but are costly to purchase and maintain and thus may not be available to many research groups. Cloud computing offers a scalable alternative here, providing near-infinite storage and on-demand processing power without the requirements of maintaining local hardware. This can be advantageous for shared resource facilities and industry environments where eliminating bottlenecks in data collection pipelines is crucial. In response to the growing adoption of cloud-based data storage, SBGrid has developed a platform specifically designed for structural biology data management and analysis in the cloud. We will discuss the challenges of managing large datasets in structural biology and strategies we have implemented for throughput, processing and storage. We will explore effective use of cloud platforms for large datasets, cost management strategies, and the benefits and limitations of cloud infrastructure for macromolecular structure determination.

